# Carriers of HLA-DRB1*04:05 have a better clinical response to abatacept in rheumatoid arthritis

**DOI:** 10.1038/s41598-023-42324-6

**Published:** 2023-09-14

**Authors:** Mariko Inoue, Yasuo Nagafuchi, Mineto Ota, Haruka Tsuchiya, Shoko Tateishi, Hiroko Kanda, Keishi Fujio

**Affiliations:** 1 Department of Allergy and Rheumatology, Graduate School of Medicine, The University of Tokyo, 7-3-1 Hongo, Bunkyo-ku, Tokyo, 113-8655 Japan; 2 Clinical Research Promotion Center, The University of Tokyo Hospital, 7-3-1 Hongo, Bunkyo-ku, Tokyo, 113-8655 Japan; 3 Department of Functional Genomics and Immunological Diseases, Graduate School of Medicine, The University of Tokyo, 7-3-1 Hongo, Bunkyo-ku, Tokyo, 113-8655 Japan; 4 Immune-Mediated Diseases Therapy Center, Graduate School of Medicine, The University of Tokyo, 7-3-1 Hongo, Bunkyo-ku, Tokyo, 113-8655 Japan

**Keywords:** Rheumatoid arthritis, Rheumatic diseases, Medical research, Rheumatology

## Abstract

HLA-DRB1 shared epitope risk alleles are the strongest genetic risk factors for rheumatoid arthritis (RA) and potential biomarkers for treatment response to biological disease-modifying antirheumatic drugs (bDMARDs). This study aimed to investigate the association between treatment response and individual HLA-DRB1 alleles in RA patients receiving different bDMARDs. We recruited 106 patients with active RA who had started abatacept, tocilizumab, or TNF inhibitors as a first-line bDMARDs. We examined the relationship between Simplified Disease Activity Index (SDAI) improvement at 3 months and HLA-DRB1 allele carriage. The results revealed that the HLA-DRB1*04:05 allele, a shared-epitope allele, was significantly associated with better SDAI improvement only after abatacept treatment (SDAI improvement 28.5% without the allele vs 59.8% with allele, *p* = 0.003). However, no significant association was found with other treatments. Both multivariate linear regression and mediation analysis confirmed that the HLA-DRB1*04:05 allele was independently associated with abatacept treatment response, regardless of anti-CCP antibody titers. The study concluded that in patients with RA receiving their first-line bDMARD treatment, carrying the HLA-DRB1*04:05 allele was associated with better SDAI improvement specifically in abatacept-treated patients. These disease-risk HLA alleles have the potential to serve as genomic biomarkers for predicting treatment response with co-stimulation blockage therapy.

## Introduction

Treatment of rheumatoid arthritis (RA) has improved dramatically with the development and approval of biologic agents (bDMARDs) and Janus kinase (JAK) inhibitors^1^. Although many bDMARDs and JAK inhibitors are now available, treatment recommendations list the two options as equivalent due to the paucity of treatment-specific predictors of response for guiding drug selection^2,3^. In practice, the choice of medication for RA is often based on the clinician's experience. In contrast, in the field of malignancy, genomic medicine has progressed, and drugs are routinely selected on a patient-by-patient basis according to the cancer genetic mutations present. In the field of autoimmune diseases, however, there is still a lack of evidence for personalized medicine. Among autoimmune diseases, RA presents a heavy burden, given the high cost of treatment and the progressive destruction of joints in the early stages of disease, and there is an urgent need to address it. Indeed, with the large number of treatment options available for RA, it seems like a plausible goal for the near future.

In predicting response to therapy for RA, the HLA gene has, to date, been the most examined genomic biomarker. The HLA-DRB1 gene is the most potent disease susceptibility gene, explaining 30–50% of the genetic risk of RA, and a specific sequence at positions 70–74 of the HLA-DRB1 allele is called a shared epitope (SE) and thought to be involved in the onset and pathology of RA^4,5^. SE positivity is associated with progressive joint destruction^6^, and it has been reported that bone destruction progresses more rapidly on X-ray when the amino acid at position 11 of HLA-DRB1 is valine^7^. Regarding the association between HLA and the efficacy of bDMARDs, a study reported a significant improvement in disease activity with TNF inhibitors when the amino acids at positions 11, 71, and 74 of HLA-DRB1 were valine, lysine, and alanine, respectively, using RA cohort data from the United Kingdom^7^. On the other hand, in 2016, an attempt was made to construct a predictive model for responsiveness to TNF inhibitors using genome-wide single nucleotide polymorphism information of RA patients, but no significant improvement in prediction accuracy was observed by adding genetic polymorphism information to the prediction model based on clinical information^8^.

Abatacept (ABT, CTLA4-Ig) is a drug that inhibits T-cell co-stimulation. There are multiple reports of good response to treatment with ABT in HLA-DRB1 SE-positive cases. In patients with RA using ABT, the SDAI remission rate was 55.3% for SE-positive patients and 20.0% for SE-negative patients, demonstrating high efficacy of ABT in SE-positive RA patients^9^. A study comparing the impact of SE on the efficacy of ABT and the JAK inhibitor tofacitinib also reported that SE was associated with DAS28 remission only in the ABT group^10^. Furthermore, in the Early-AMPLE study, a prospective study directly comparing ABT with the TNF inhibitor adalimumab, ABT was more effective in SE-positive patients in terms of American College of Rheumatology core set and DAS28 remission^11^. Although these reports suggest that SE alleles are strongly associated with treatment response, especially in ABT, details on the specific HLA-DRB1 allele associated with susceptibility are scarce.

In this study, we investigated the association between treatment response and individual HLA-DRB1 alleles in patients with RA using ABT, tocilizumab (TCZ, an IL-6 receptor inhibitor), or a TNF inhibitor as their first bDMARD, and attempted to identify HLA alleles associated with treatment outcome.

## Methods

### Patients

Japanese patients with RA who had received their first bDMARD between June 2012 and August 2018 in the Tokyo University Biologics Registry for RA (TOBIRA) and continued it for at least 3 months were included. All patients fulfilled the 1987 ACR^12^ or 2010 ACR/EULAR classification criteria for RA^13^. The selection of bDMARDs was made based on the judgment of both the physician and the patient. From the revised version in 2013 to the latest revised edition in 2022, EULAR recommendations for the management of RA have been listed TNF inhibitor, IL-6 inhibitor, and abatacept equally as bDMARDs^14,15^, and treatment selection was made with reference to these recommendations. Patients who had been previously treated with bDMARDs or JAK inhibitors were excluded, and those who met SDAI remission (≤ 3.3) at the start of their first bDMARD regimen were excluded. The study was approved by the ethics committee of the University of Tokyo (no. 11592). All methods were carried out in accordance with Declaration of Helsinki and Japanese government’s Ethical Guidelines for Medical and Health Research Involving Human Subjects.

### Clinical data collection

We evaluated age, sex, disease duration, smoking history, Anti-CCP (cyclic citrullinated peptide) antibody, rheumatoid factor, and concomitant methotrexate (MTX) and oral prednisolone treatment at baseline. Anti-CCP antibody titer was measured using the STACIA MEBLux Test CCP, which is approved as an in vitro diagnostic medical device in Japan. The measurement range of this test kit is 0.6–500 U/mL. Oral glucocorticoid dose was converted to prednisolone equivalent dose. The following variables were evaluated at baseline and at 3 months of bDMARD treatment: tender joint count (TJC) and swollen joint count (SJC) in 28 joints, patient global assessment (PtGA), evaluator global assessment (EGA), and blood CRP level. The CRP level was measured as mg/dl. Global health (GH) was customarily replaced by the PtGA in millimeters on a visual analog scale. Scores for the PtGA and EGA were measured in centimeters on a 0–100 mm visual analog scale. We determined the SDAI score using the following equation^16^:

$${\text{SDAI }} = {\text{ SJC28 }} + {\text{ TJC28 }} + {\text{ PtGA }} + {\text{ EGA }} + {\text{ CRP}}$$

Improvement in disease activity at 3 months was assessed based on change of SDAI value from baseline^17^:

$$\left( {{\text{SDAI at baseline}}{ - }{\text{SDAI at 3}} {\text{months}}} \right)/{\text{SDAI at baseline}} \times {1}00 \, [\% ].$$

DAS28-CRP was calculated following the definition:

$$\text{DAS28-CRP} = \, 0.{56}*\surd \left( {{\text{TJC28}}} \right) \, + \, 0.{28}*\surd \left( {{\text{SJC28}}} \right) \, + \, 0.0{14}*{\text{GH }} + \, 0.{36}*{\text{ln}}\left( {{\text{CRP}} + {1}} \right) \, + \, 0.{96}$$

### HLA allele and treatment responsiveness

HLA-DRB1 alleles were determined by next generation sequencing using peripheral blood of patients. We examined ten alleles with an allele frequency exceeding 0.03 based on the participants in this study. Of them, HLA-DRB1*01:01, *04:05, and *04:10 were defined as SE^18^.

We used the percent change in SDAI from baseline to 3 months after each treatment as an assessment measure for treatment responsiveness for the following reasons. Tocilizumab blocks the IL-6 receptor and inhibits the production of acute-phase reactants, including ESR and CRP. Since the weight of ESR and CRP levels is quite high in the DAS28 formula^19,20^, the remission rate of patients under tocilizumab treatment was reported to be higher when using DAS28 compared to SDAI^21^. In this study, we evaluated the treatment responsiveness among bDMARDs, including tocilizumab, and therefore, we considered that using SDAI for evaluation was appropriate. The ACR20 has been validated as the best discriminator of efficacy in placebo-controlled trials. However, when assessing depth of response with respect to disease activity, continuous scales, such as SDAI is commonly employed. Smolen et al. investigated SDAI improvement rates in previous large-scale clinical trials and found that these rates serve as sensitive treatment response criteria. They also observed that optimal cutoffs for SDAI improvement rates vary across different trials^22^. Given the limited number of cases in our study, we utilized the SDAI improvement rate as a more sensitive continuous variable to investigate the relationship between HLA haplotypes and treatment response.

The percent change in SDAI from baseline to 3 months after each treatment was examined, and the difference in percent change between carriers and non-carriers of these HLA alleles was analyzed with a Welch's *t* test, followed by a multiple testing correction with the Benjamini–Hochberg method. We applied a significance threshold for multiple comparisons based on the Benjamini–Hochberg method with a false discovery rate (FDR) of < 0.05. Dose effects based on HLA-DRB1 alleles were not tested because of the limited number of study participants.

### Univariate and multivariate linear regression analysis of abatacept response

Thirty-seven patients who underwent ABT treatment were included in the analysis. A univariate linear regression analysis was performed using the SDAI improvement rate after 3 months as the objective variable and each clinical item and the HLA-DRB1*04:05 allele were the explanatory variables. To account for the varying scales of the explanatory variables, we normalized both the objective and explanatory variables. This normalization process aimed to ensure that all variables had a mean of zero and a variance of one. We further conducted multiple regression analysis using the HLA-DRB1*04:05 allele, disease duration, and the anti-CCP antibody titer as explanatory variables. These variables were selected based on their significance (*p* < 0.20) in the univariate analyses. The response variable in this analysis was the SDAI improvement rate at 3 months.

### Mediation analysis

Thirty-seven ABT-treated patients were included in the mediation analysis. The relationships between the HLA-DRB1*04:05 allele (negative or positive), anti-CCP antibody titer, and SDAI improvement rate at 3 months were tested by mediation analysis. The SDAI improvement rate at 3 months was treated as the dependent variable of the linear regression model. The HLA-DRB1*04:05 allele was treated as the independent variable and anti-CCP antibody titer was treated as the mediator. To account for the varying scales of the variables, we normalized all variables. *p* values were calculated via 1000-time bootstrapping.

### Statistical analysis

All statistical tests were performed using GraphPad Prism v9.3.1 (GraphPad Software) and R v4.1.3 (The R foundation). Mediation analysis was performed using the R mediation package v4.5.0.

### Ethics approval and consent to participate

This study was approved by the ethics committee of the University of Tokyo (11592), and informed consent was obtained from all participants included in the study.

## Results

### Patient characteristics and HLA alleles

The study included 106 patients with RA who had active disease and were initiating their first bDMARD (Supplementary Figure S1). Table 1 shows the patient characteristics. The bDMARDs used in this study included 37 patients with ABT, 28 with TCZ, and 41 with TNF inhibitors. There were no significant differences in SDAI disease activity at the start of bDMARD treatment or 3 months after treatment (Kruskal–Wallis test, *p* = 0.63 at baseline, *p* = 0.75 at 3 months). ABT patients were older (one-way analysis of variance, *p* < 0.0001) and TNF inhibitors patients were more likely to use MTX (Chi-square test, *p* = 0.014). This study includes patients who initiated bDMARDs with SDAI low disease activity. These patients began bDMARDs with the goal of tapering glucocorticoids, or their treatment was intensified with bDMARDs because their disease activity was considered moderate when evaluated using disease activity measures other than SDAI.

**Table 1:** Clinical characteristics of patients.

Characteristic	Abatacept (n = 37)	Tocilizumab (n = 28)	TNF inhibitor (n = 41)	*p*
Age, years, mean ± SD	68.0 ± 8.3	56.4 ± 12.6	59.2 ± 12.1	< 0.0001^§^
Sex, female, %	86.5	82.1	82.9	0.87^‡^
Disease duration, years	6.4 (2.9–20.6)	5.6 (1.3–12.3)	4.4 (1.0–13.3)	0.24^†^
Anti-CCP antibody positive, %	86.5	67.9	80.5	0.18^‡^
Anti-CCP antibody titer (U/mL)	102 (17.2–333)	25.9 (0.9–143)	100 (10.8–230)	0.20^†^
RF positive, %	83.8	64.3	80.5	0.15^‡^
RF titer (IU/mL)	75.0 (21.5–131)	38.0 (7.3–131)	56.0 (20–148)	0.44^†^
Smoking, %	27.0	46.4	29.3	0.21^‡^
Methotrexate user, %	56.8	60.7	85.4	0.014^‡^
Methotrexate dose (mg)	6.0 (0.0–8.0)	7.0 (0.0–10.0)	10.0 (6.0–10.0)	0.004^†^
Oral prednisolone user, %	64.9	60.7	61.0	0.92^‡^
Oral prednisolone dose (mg)	4.0 (0.0–5.0)	3.0 (0.0–5.0)	3.0 (0.0–5.0)	0.54^†^
Baseline DAS28-CRP, mean ± SD	4.4 ± 1.5	4.0 ± 1.4	4.2 ± 1.1	0.52^§^
Baseline SDAI	18.7 (11.9–38.3)	16.6 (11.7–25.5)	20.5 (13.5–28.6)	0.63^†^
Baseline SDAI Remission/LDA/MDA/ HDA	0/5/19/13	0/5/17/6	0/8/21/12	0.80^♦^
3 months DAS28-CRP, mean ± SD	3.3 ± 1.4	2.7 ± 1.1	2.9 ± 1.1	0.11^§^
3 months SDAI	10.1 (4.8–16.9)	8.5 (5.2–14.0)	8.3 (4.8–17.0)	0.75^†^
3 months SDAI Remission/LDA/MDA/ HDA	5/15/13/4	4/13/9/2	7/15/17/2	0.85^♦^

Values are the median (first quartile–third quartile) unless indicated otherwise.

*CCP* cyclic citrullinated peptide; *RF* rheumatoid factor; *DAS28-CRP* DAS28 using the CRP; *SDAI* simplified disease activity index; *LDA* low disease activity; *MDA* moderate disease activity; *HAD* high disease activity.

^†^Based on Kruskal–Wallis test.

^‡^Comparisons between the 3 groups are based on Chi-square test.

^§^Based on one-way analysis of variance.

^♦^Fisher’s exact test was performed for the proportion below LDA.

There were no significant differences in allele frequencies of the major HLA-DRB1 alleles between the ABT, TCZ, and TNF inhibitors groups (Table 2).

**Table 2:** HLA-DRB1 allele frequencies.

HLA alleles	Abatacept (n = 37)	Tocilizumab (n = 28)	TNF inhibitor (n = 41)	*p*‡
Total	74 (100)	56 (100)	82 (100)	
DRB1*01:01 #	5 (6.8)	3 (5.4)	7 (8.5)	0.77
DRB1*04:05 #	17 (23)	9 (16.1)	20 (24.4)	0.48
DRB1*04:06	2 (2.7)	5 (8.9)	3 (3.7)	0.21
DRB1*04:10 #	3 (4.1)	2 (3.6)	4 (4.9)	0.93
DRB1*08:03	5 (6.8)	4 (7.1)	6 (7.3)	0.99
DRB1*09:01	10 (13.5)	10 (17.9)	15 (18.3)	0.69
DRB1*12:01	3 (4.1)	2 (3.6)	2 (2.4)	0.85
DRB1*13:02	3 (4.1)	1 (1.8)	4 (4.9)	0.64
DRB1*15:01	6 (8.1)	0 (0)	3 (3.7)	0.072
DRB1*15:02	3 (4.1)	7 (12.5)	9 (11)	0.18
Other DRB1 alleles	17 (23.0)	13 (23.2)	9 (11.0)	0.086

All data are shown as n (%).

^‡^ Comparisons between the 3 groups are based on Chi-square test.

^#^ Shared epitope alleles.

### Treatment response by HLA allele

In each of the 3 treatment groups, treatment response 3 months after bDMARD initiation was compared between carriers and non-carriers of the major HLA-DRB1 alleles (Table 3). Of all the HLA-DRB1 alleles, the only one that showed a significant difference in percent change in SDAI after 3 months was HLA-DRB1*04:05, one of the SE alleles in ABT use (28.5% SDAI improvement in HLA-DRB1*04:05 allele non-carriers and 59.8% SDAI improvement in HLA-DRB1*04:05 allele carriers, *p* = 0.003, false discovery rate = 0.039). In other words, among the SE alleles, HLA-DRB1*04:05 was a clear predictor of good prognosis among those treated with ABT.

**Table 3:** Effects of HLA alleles on SDAI improvement after treatment.

HLA alleles	Number of patients without allele	Number of patients with allele	Mean SDAI improvement without allele (%)	Mean SDAI improvement with allele (%)	*p*†	Benjamini–Hochberg adjusted *p*
Abatacept (n = 37)						
DRB1*01:01#	32	5	41.2	53.4	0.449	0.662
DRB1*04:05#	20	17	28.5	59.8	0.003*	0.039*
DRB1*04:06	35	2	45.0	5.5	0.654	0.662
DRB1*04:10#	34	3	44.3	27.0	0.662	0.662
DRB1*08:03	33	4	41.4	55.3	0.320	0.662
DRB1*09:01	30	7	47.0	25.0	0.185	0.662
DRB1*12:01	34	3	43.5	35.3	0.558	0.662
DRB1*13:02	34	3	43.4	36.7	0.657	0.662
DRB1*15:01	31	6	47.7	17.8	0.237	0.662
DRB1*15:02	34	3	44.7	22.0	0.446	0.662
Tocilizumab (n = 28)						
DRB1*01:01#	25	3	43.1	42.3	0.974	0.974
DRB1*04:05#	19	9	47.2	34.2	0.521	0.759
DRB1*04:06	23	5	47.5	22.4	0.447	0.759
DRB1*04:10#	26	2	41.7	60.5	0.552	0.759
DRB1*08:03	24	4	42.3	47.5	0.853	0.938
DRB1*09:01	19	9	47.8	33.0	0.244	0.759
DRB1*12:01	26	2	41.4	64.5	0.292	0.759
DRB1*13:02	27	1	42.7	52.0	–	–
DRB1*15:01	28	0	43.0	–	–	–
DRB1*15:02	21	7	40.8	49.9	0.541	0.759
TNF inhibitors (n = 41)						
DRB1*01:01#	34	7	50.4	40.4	0.587	0.893
DRB1*04:05#	23	18	44.4	54.2	0.313	0.893
DRB1*04:06	38	3	48.4	53.0	0.910	0.910
DRB1*04:10#	37	4	48.4	52.0	0.756	0.893
DRB1*08:03	35	6	51.0	35.3	0.288	0.893
DRB1*09:01	27	14	52.0	42.3	0.345	0.893
DRB1*12:01	39	2	47.5	72.5	0.375	0.893
DRB1*13:02	37	4	47.8	57.5	0.601	0.893
DRB1*15:01	38	3	48.2	55.7	0.671	0.893
DRB1*15:02	33	8	49.5	45.3	0.704	0.893

^*^p < 0.05.

^#^Shared epitope alleles.

^†^Based on Welch two-sample *t* tests.

### HLA-DRB1*04:05 and treatment response in ABT use

In the Japanese ACPA-positive RA population, the HLA-DRB1*04:05 allele has a deep impact on RA pathogenesis, with an odds ratio of 5.0 compared to healthy individuals^23^. Since our HLA allele analysis showed a specific prognostic effect of the HLA-DRB1*04:05 allele on ABT treatment (Table 3), we continued to focus on the effect of this allele on ABT treatment response.

**Figure 1:**
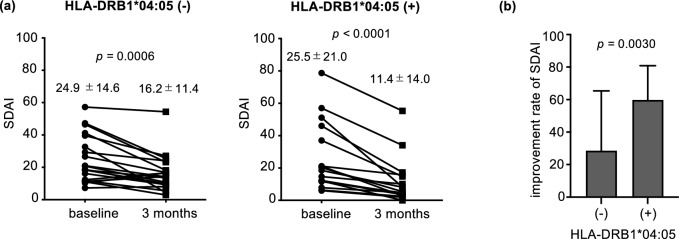
The HLA-DRB1*04:05 allele is associated with better response to abatacept treatment. (**a**) SDAI disease activity at baseline and 3 months after ABT treatment (HLA-DRB1*04:05 non-carrier, n = 20 and HLA-DRB1*04:05 carrier, n = 17; *t* test). (**b**) Comparison of SDAI improvement rate after 3 months of ABT treatment, stratified by the HLA-DRB1*04:05 carriage. *t* test.

Figure 1a shows the trend of SDAI between HLA-DRB1*04:05 carriers and non-carriers during ABT treatment. Three months after the start of treatment, SDAI significantly improved regardless of HLA-DRB1*04:05 status in the ABT group. However, the rate of improvement was significantly higher in HLA-DRB1*04:05 carriers (Fig. 1b,t test, *p* = 0.0030).

Also, we investigated the association between the presence or absence of the HLA-DRB*04:05 haplotype and the achievement rate of SDAI50 in patients treated with ABT. SDAI 50 is defined as a 50% improvement in SDAI and was proposed by Alehata as a measure that correlates with ACR20, which is considered the gold standard for evaluating treatment response in clinical trials^24^. It is worth noting that SDAI50 is also a measure that correlates with EULAR response, which is commonly used to assess treatment response in routine clinical practice.

The results revealed that the SDAI50 achievement rate was 70.6% in patients with the DRB1*04:05 haplotype, compared to 30.0% in those without the DRB1*04:05 haplotype (*p* = 0.022, Fisher’s exact test).

Also, we investigated the differences in SDAI components based on the presence or absence of HLA-DRB1*04:05. We examined TJC28, SJC28, PtGA, EGA and CRP at baseline and after ABT treatment in relation to HLA-DRB1*04:05 carriers and non-carriers (Supplementary Figure S2). Regarding TJC28 and PtGA, we did not observe significant improvement after ABT treatment in HLA-DRB1*04:05 non-carriers. However, HLA-DRB1*04:05 carriers showed improvement in all SDAI components, including TJC and PtGA, after ABT treatment.

From these findings, it is concluded that the presence of the HLA-DRB1*04:05 haplotype is associated with better SDAI improvement in patients treated with ABT.

### Comparison of HLA-DRB1*04:05 and ACPA as predictors of ABT treatment response

To examine the factors involved in ABT response to treatment, linear regression analysis was performed on the rate of SDAI improvement after 3 months of treatment (Table 4). In the univariate analysis, being a carrier of HLA-DRB1*04:05 was significantly associated with an increased rate of SDAI improvement (standardized partial regression coefficient β = 0.46, *p* = 0.0039), but other clinical parameters were not significant prognostic factors. We further conducted multiple regression analysis using the HLA-DRB1*04:05 allele, disease duration, and the anti-CCP antibody titer as explanatory variables. These variables were selected based on their significance (*p* < 0.20) in the univariate analyses. As a result, carriage of HLA-DRB1*04:05 was associated with ABT efficacy independently of ACPA titer (β = 0.48, *p* = 0.0052).

**Table 4:** Linear regression analysis of 3-month SDAI improvement rate after abatacept treatment.

Variable	Univariate analysis			Multivariate analysis		
	Standardized β	95%CI	*p*	Standardized β	95%CI	*p*
Age (year)	− 0.04	− 0.39, 0.3	0.8			
Male sex	0.22	− 0.12, 0.55	0.2			
Disease duration (year)	− 0.25	− 0.58, 0.09	0.14	− 0.28	− 0.59, 0.02	0.06
Anti− CCP antibody titer (U/mL)	0.25	− 0.09, 0.58	0.14	0.03	− 0.29, 0.36	0.84
RF titer (IU/mL)	0.01	− 0.33, 0.35	0.95			
Smoking	− 0.13	− 0.47, 0.21	0.43			
Methotrexate dose (mg)	− 0.10	− 0.44, 0.24	0.54			
Prednisolone dose (mg)	− 0.16	− 0.5, 0.17	0.33			
Baseline DAS28-CRP	0.08	− 0.27, 0.42	0.65			
Baseline SDAI	0.07	− 0.28, 0.41	0.69			
HLA-DRB1*04:05	0.46	0.16, 0.77	0.0039*	0.48	0.15, 0.8	0.0052*

*CCP* cyclic citrullinated peptide; *RF* rheumatoid factor; *DAS28-CRP* DAS28 using the CRP; *SDAI* simplified disease activity index; *95% CI* 95 percent confidence interval.

SDAI improvement rate is calculated as (SDAI at baseline−SDAI at 3 months)/SDAI at baseline × 100 [%] and maximal score of SDAI improvement rate is 100.

To account for the varying scales of the explanatory variables, we normalized both the objective and explanatory variables. This normalization process aimed to ensure that all variables had a mean of zero and a variance of one.

*p < 0.05.

**Figure 2:**
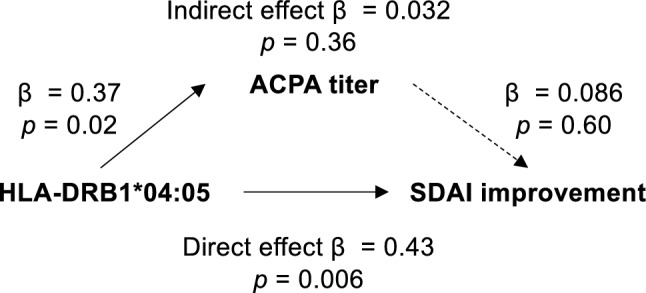
The HLA-DRB1*04:05 allele is directly linked to abatacept treatment response. A mediation analysis of the relationships between the HLA-DRB1*04:05 allele carriage, anti-CCP antibody titer, and the SDAI improvement rate 3 months after ABT treatment (n = 37). Solid lines represent significant associations, while the dashed line indicates a non-significant association.

To further clarify the relationship between the HLA-DRB1*0405 allele, ACPA titer, and SDAI improvement rate, a causal mediation analysis was performed. The HLA-DRB1*04:05 allele was directly associated with the rate of SDAI improvement, rather than through an indirect effect on ACPA titer (Fig. 2). These results indicate that HLA-DRB1*04:05 is more directly associated with ABT prognosis than ACPA titer.

## Discussion

In this study, we showed that among the SE alleles, HLA-DRB1*04:05 in particular was strongly associated with ABT treatment prognosis. The allele frequency of the HLA-DRB1*04:05 in Japanese patients with ACPA-positive RA is reported to be about 28%. Since each individual carries two HLA-DRB1 alleles, approximately half of the ACPA-positive RA patients have at least one copy of HLA-DRB1*04:05. And HLA-DRB1*04:05 is strongly associated with the development of ACPA-positive RA, having an odds ratio of 5.0^23^. HLA, being innate and unchanging throughout a person's lifetime, suggests that the association between HLA and treatment prognosis is not merely coincidental. In other words, the HLA genotype is the cause, leading to favorable treatment outcomes. Although several associations between SE and ABT efficacy have been reported^9–11^, details at the allele level are limited, even though the significance of the specific alleles as potential biomarkers is promising.

In this study, it was found that only HLA-DRB1*04:05 demonstrated an association with the responsiveness to ABT treatment, while HLA-DRB1*01:01 and 04:10, which share similar SE, did not show a significant association with treatment responsiveness. In addition to the effect of the small sample size, the following reasons can be considered. Amino acids at positions 11, 13, and 67 of HLA-DRB1, which are amino acid sequences other than SE, are also implicated in the risk of developing RA. Specifically, it has been found that in DRB1*04:05 and 04:10 the valine at position 11 is the amino acid most strongly associated with RA susceptibility, whereas DRB1*01:01 has a different amino acid, leucine, at position 11^25^. Additionally, in a study reporting on the risk of developing RA among the Japanese population, it has been demonstrated that the risk of RA differs based on the variant of HLA-DRB1, even sharing the same HLA SE allele. It is suggested that HLA-DRB1*01:01, 04:05, and 04:10 are not biologically equivalent^23^. Furthermore, it has been reported that HLA risk alleles for autoimmune diseases significantly impact the pattern of CDR3 sequences in T-cell receptors. Additionally, CDR3 sequences modified by HLA risk alleles have been associated with the recognition of citrullinated antigens. Therefore, it is believed that sequences other than SE are also associated with the development and progression of RA and other diseases^26^.

SE and ACPA-positive RA are strongly associated, and ACPA is also associated with ABT treatment prognosis^27,28^. Previous reports have also shown that SE is associated with ABT outcomes, even after adjusting for the effect of ACPA^9,10^. In this study, both multiple regression analysis and mediation analysis suggested that the effect of the HLA-DRB1*04:05 allele was not an indirect effect mediated by ACPA (Table 4, Fig. 2). The impact of SE has been reported to be stronger in ACPA-positive RA than in ACPA-positive non-RA controls^29,30^. In other words, SE may be involved in the onset of RA through mechanisms other than direct effects on ACPA positivity. RA-risk HLA is robustly associated with the T-cell receptor repertoire of CD4^+^ T cells^26,31^. RA-sensitive HLA alleles, such as HLA-DRB1*04:05, are associated with autoreactive CD4^+^ T cells, which may be therapeutic targets for ABT.

In this investigation, the use of methotrexate was low in the abatacept group. Because, in general, it was reported that concomitant use of MTX may not augment the effectiveness of ABT. For example, in a phase III study, ABT did not elicit immunogenicity associated with loss of safety or efficacy, either with or without MTX^32^. Also, in a retrospective cohort study, in RA patients with similar background characteristics undergoing abatacept treatment, concomitant MTX did not seem to affect clinical outcomes^33^. Based on these findings, we believe that ABT would be a suitable treatment option in daily clinical practice in patients with contraindications to MTX.

In this study, the association between the HLA-DRB1*04:05 allele, an SE allele, and favorable treatment outcomes was significant only in ABT-treated patients, but not in those treated with the IL-6 receptor inhibitor TCZ or a TNF inhibitors. This is consistent with the association between the better prognosis with ABT and SE reported in the Early-AMPLE trial comparing ABT with the TNF inhibitor adalimumab^11^. SE was not strongly associated with efficacy of the JAK inhibitor tofacitinib either^10^. These findings may reflect the difference in mechanism of action between ABT, which inhibits co-stimulation of antigen-presenting cells and CD4^+^ T cells, and IL-6 receptor inhibitors, TNF inhibitors, and JAK inhibitors, which are drugs that block inflammatory cytokine signaling.

There are several limitations to this study. First, because of the retrospective nature of this analysis, we cannot exclude the possibility of selection bias. Second, the number in each treatment group is small, so the effect of HLA alleles with a small frequency or small effect size may not have been fully realized. Third, since this study was conducted in a single Japanese cohort and there are ethnic differences in HLA-DRB1 allele frequencies, it is necessary to verify whether the results can be generalized to other cohorts, including other ethnic groups.

In conclusion, we analyzed the association between HLA-DRB1 alleles and prognosis in Japanese patients with RA who were starting ABT, TCZ, and TNF inhibitor treatment, and we showed that among SE alleles, the HLA- DRB1*04:05 allele was associated with better outcomes with ABT. This study demonstrates the possibility of stratifying RA patients by disease-risk HLA alleles, and supports the need for a larger prospective study.

## Data Availability

The datasets used and analyzed during the current study are available from the corresponding author on reasonable request. We cannot share raw HLA allele information of the patients publicly because it can be considered as personal information in Japanese regulations. Summary data of the clinical information and HLA allele frequencies are provided as Table 1 and Table 2. We used publicly available software for the analyses. Custom codes are available from the corresponding author on reasonable request.

## Abbreviations

RA: Rheumatoid arthritis
bDMARDs: Biological disease-modifying antirheumatic drugs
SDAI: Simplified disease activity index
JAK: Janus kinase
SE: Shared epitope
ABT: Abatacept
TCZ: Tocilizumab
CCP: Cyclic citrullinated peptide
MTX: Methotrexate
TJC: Tender joint count
SJC: Swollen joint count
PtGA: Patient global assessment
EGA: Evaluator global assessment
GH: Global health
ACPA: Anti-citrullinated protein antibodies
